# An Analysis of Characteristics of Children’s Growth through Practical Art

**DOI:** 10.3390/healthcare8020109

**Published:** 2020-04-24

**Authors:** Lan Yu, Yukari Nagai

**Affiliations:** School of Knowledge Science, Japan Advanced Institute of Science and Technology, Ishikawa, Nomi City 9231211, Japan; yulan@jaist.ac.jp

**Keywords:** children’s growth concern, children’s painting learning, children’s painting features, digital image analysis

## Abstract

Children’s paintings reflect their growth environments and psychological conditions, and these growth environments represent children’s family and educational environments in primary schools. The characteristics of these paintings change as children grow, and children’s expressiveness in the paintings also improves. Children’s paintings are a representation of their perceptions of things; children transform their perceptions into images that can be understood and observed by people. This research studies the growth characteristics of children based on professional painting techniques. A digital image analysis method was used to analyze the painting techniques of children aged between 7 and 13. The growth characteristics of the different age groups were combined to analyze the representative characteristics of children’s paintings at different ages. Lastly, the results of part of a questionnaire survey were used to assist in studying these characteristics. Analysis of these paintings shows that children have a poor ability to control the scale of the objects. Furthermore, the details of the objects are ignored, and children have a poor imitation ability. Young children have lower spatial cognitive abilities than older children, and girls prefer to participate in painting more than boys.

## 1. Introduction

Paintings are endowed with individual creative ideas by children. Painting is one of the ways to represent people’s imagination [1] (pp. 11–29); in other words, people like to record their imaginary and observed scenes in paintings. Moreover, paintings are the sum of people’s perceptions of objects (such as the perceptions of the appearance of the shape, volume, and color of the object). According to the content of children’s paintings, teachers can learn about their living environment [2,3]. Children’s paintings contain features of the described objects and indicate the children’s observation ability [4]. Children will record what they see (such as life scenes, activity scenes, and natural scenes) in paintings, and their observation ability can also be shown in the details of the paintings (such as depictions of the decorative patterns of objects, facial expressions of characters, and figural postures). The positional, distance, and proportional relationships of different objects in paintings show the impact of events or people on children [5]. The content of children’s paintings is derived from their life and painting experiences [6]. There is an existential communication among color, taste, touch, smell, sound, and weight in artwork, which constitutes its overall content [7]. The same intrinsic link exists in the main components of paintings: There is a connection between color, content, composition, and lines in paintings, and the content and structure of the painting determine its quality. When guiding children to create paintings, fine art teachers should fully understand the content of children’s paintings (such as color distribution, the layout of the lines, and shapes of objects in paintings) and accurately determine the technical problems in these paintings. These are key to helping children to improve their painting ability. Therefore, analyzing the content of children’s paintings helps fine art teachers to guide children more effectively to create them.

In the past, research on children’s paintings relied on the human visual system to judge them. This kind of painting evaluation method is affected by personal emotions, cultural background, living environment, and preferences, and is also influenced by personal subjective evaluation standards. However, using digital image analysis methods can reduce experimental error caused by personal subjective evaluation, as the results obtained from such methods are more objective. Paintings can be evaluated from extracted image features from the perspective of local and global features to determine visual quality [8,9]. This method can help people to simply assess the visual quality of paintings. Assessment (including, e.g., lines, color features, and composition features) involves dividing the image according to different details and then using a digital image analysis method to evaluate said details [10]. In this study, children’s paintings are converted into digital images, and an image binarization method is used to analyze the images. The analysis process of image binarization selects the best threshold in the image by using the Otsu algorithm, and then retains the image’s texture features required for research purposes [11,12], [13] (pp. 445–448). Each color in a digital image has a corresponding pixel value; the image binarization method sets the gray value of pixels in the image to 0 or 255 [14]. Using a digital image analysis method to analyze children’s paintings can help art teachers to analyze them more accurately and thus to effectively reduce experimental error.

Painting technique training is necessary for children’s painting education [15]. For example, training in the use of painting tools can help children create different styles of paintings, and imitation training can help children understand the process and method of painting creation. Learning the rules of painting composition can help children improve the visual effects of their paintings. In the process of painting practice for children who have not received professional drawing training, due to the lack of clear painting training goals and evaluation standards, the drawing ability of children cannot be improved [16] (pp. 47–65). This also highlights problems (such as composition and modeling problems) when applying painting techniques in the paintings of children who have not received professional painting training. Therefore, this study evaluates the paintings of children who are not trained and then discusses existing problems while children create paintings. 

The content of a painting determines its quality, and composition, lines, colors, and shapes represent this quality [17]. Content analysis can help teachers to determine the features of high-quality paintings; in terms of children’s paintings, the basic characteristics include consistency of tones, relevant content, and a sense of space [18]. These factors can also assist the learner in improving painting ability. In addition, studying the common characteristics of children’s paintings can help fine art teachers better understand and observe children’s painting habits, such as painting styles, material use, and creative habits. In the process of guiding children to paint, fine art teachers need to consider children’s learning and development. After fully understanding their painting habits, they can then make teaching plans according to the characteristics of children’s paintings. This can also help fine art teachers to acquire knowledge points that children cannot grasp in the process of learning how to paint.

Children of different ages have different abilities in terms of painting expression, cognition of painting creation, application ability of painting technology, and arrangement of picture content. This study used a fixed painting style (determined beforehand) to test children. Each child was required to complete a realistic painting work and had to create a painting with the same painting theme—which involved a description of a scene (such as a life scene, a nature scene, or an event) and characters (such as friends, family members, or classmates). A fixed painting theme was used to compare paintings of children of different ages and genders in later data statistics. This also reduced the difficulty in analyzing the content of the paintings and improved the reliability of the research results. Studying children’s paintings of different ages under the same theme can also help teachers to determine different features of children’s paintings.

## 2. Background

The goal of children’s painting education is to create and develop children’s abilities in beauty cognition. The process by which children learn to create paintings is also a process that helps children understand and experience beauty. Painting creation can cultivate children’s sense of innovation, esthetic judgment, and ability to evaluate beauty. Fine art education affects students’ thinking styles [19], and helps them to improve their imagination, creativity, and observation in the process of painting [20]. Fine art education also affects students’ observation ability [21]; for example, after children learn about perspective, they have the cognitive ability of space. When students have learned basic painting techniques, they start to pay attention to the details of objects in their paintings—for example, the decorative patterns, proportions, distances, and positional relationships of objects. After children learn to paint, they also start to pay attention to the proportional relationships between objects in their paintings [22]. In Asian nations (including China, Hong Kong, Japan, Taiwan, South Korea, and Singapore), many preschool children often find themselves in learning environments of scripted teaching, rote learning, and standardized assessment [23,24,25]. Fixed-mode education can make children lose their sense of innovation, and rote learning can also result in a loss of interest in learning [26]. Therefore, changing education of fixed patterns can help children increase their interest in learning. Considering children’s artistic cognitive ability from the perspective of their painting habits, their drawing skills, and the application of painting techniques, Malchiodi (1998) combined different psychological research methods to evaluate the lines, shapes, sizes, positions, and colors of objects in children’s paintings. It is important to cultivate children’s composition ability in order to help them to arrange objects in paintings in the proper position. The purpose of teaching children painting composition is to help them to establish the cognition of the positional relationship of objects and perspectives, and to help them to structure paintings to well-defined levels (the proportional relationship of different objects in paintings).

Art education itself has important implications for human growth and development [27]. Children’s drawing ability is related to their cognitive ability [28]. Children’s paintings develop from a simplification to a complex representation of an object’s shape [29]. When younger children first start painting, they are less expressive [5]; therefore, their paintings show simple shapes and lack details, and most of the objects in the paintings are composed of lines. With the development of their cognitive ability and continuous painting practice, children begin to use different painting materials to create different styles of painting. The improvement of children’s drawing ability is achieved by the guidance of art teachers and the study of art courses [30], which refers to the theory of “Discipline-Based Art Education (DBAE).” The aim of DBAE is to help children to create esthetic perception paintings through systematic fine art courses. For children, painting training courses have a positive impact on their painting abilities. The significance of art education is not only to improve children’s ability to paint, but also, and more importantly, to help children develop a sense of innovation. The cultivation of children’s imagination is more important than their knowledge education, as imagination can promote the progress of knowledge. Learning activities (such as learning music, handmade crafts, and painting) can better help children learn in the process of acquiring knowledge, as well as build a link between children’s learning at school and their daily experiences [31]. A study about science education mentions that fine arts play a decisive role in science education [32], and further studies have shown that images are more likely to evoke human imagination than words [33]. The results of art education directly determine the results of the cultivation of children’s innovative consciousness. In the process of painting creation, the painting construction process also enhances children’s esthetic consciousness. In addition, researchers have proved that fine arts can efficiently promote the learning of other scientific knowledge [34].

Paintings contain both things in real life and things approaching real life [33]. Artworks combine all possible connected things within a valid range, and analyzing the content of paintings through scientific analysis methods is a way of analyzing the creative process and the creative consciousness of the creators. People have the talent to classify according to the weight, color, size, function, or shape of things. In the process of painting creation, differences in things can be distinguished by changes in these lines, colors, and shapes of objects. When people observe the object, image information transmitted by the object is immediately combined with the subjective consciousness of human beings; this is the exchanging process of image information. A painting is a superficial image of the combination of the human subjective consciousness and the information conveyed by objects. Painting creation is closely connected to people’s living environment. The process of children’s painting creation is also the process by which children reproduce a scene that they observe (such as a natural scene, a life scene, or a school scene) [35]. The role of painting education is to combine children’s observation experience with teachers’ painting skill guidance in order to help children better complete painting creation. Painting training can help children to consider the relationship between people and the natural environment [36,37]. Children’s paintings reflect their esthetic consciousness, and ideal painting training methods can help children to improve their drawing skills.

Children’s painting researcher Viktor Lowenfeld divided the development of children’s painting into six stages: The “scribble stage” (2–4 years old), the “pre-schematic stage” (4–7 years old), “schematic stage” (7–9 years old), the “dawning realism stage” (9–11 years old), the “pseudo-realism stage” (11–13 years old), and the “decision stage (the adolescent period)” [38]. Children’s paintings involve their imaginative, expressive, and creative abilities [39] (pp. 194–225), and the development of their paintings is related to their cognitive ability [40]. The paintings of children of different ages reflect the development of their understanding of visual information; in particular, the content of children’s paintings is related to the characteristics of their psychological development [36]. Thus, children’s paintings need to be analyzed in combination with their painting performance at different ages. The study of children’s painting education has predominantly focused on the composition of their paintings, and psychological analysis methods have been used to study the content of these paintings. Analyzing children’s paintings is important as it can help teachers to determine any problems during the process of learning how to create paintings. The research also shows that art can help children to develop different cognitive skills; for example, art can help children to develop socio-emotional and motor skills. Moreover, art has a positive impact on children’s self-expression [41].

## 3. Method

### 3.1. Content for the Research

This research was conducted by utilizing questionnaires and digital image analysis. The questions included in the questionnaire were based on painting guidelines and children’s preferences for painting creation (Appendix A). The role of the questionnaire was to determine whether the children participating in the survey met the requirements of the study, since the participants in this study were children who had not received professional painting training. Children have a fixed painting creation mode after they receive painting training [42]. For instance, children arrange people or objects in a fixed position (Question 1 in the questionnaire) or decorate paintings with fixed patterns and color (Question 2 in the questionnaire). The training of painting skills includes imitation training (Question 3 in the questionnaire) and composition training (Question 5 and 6 in the questionnaire) [27]. Thus, inquiring about children’s imitation ability and their ability to control objects in paintings is helpful for further judgments about whether children have received painting training or not. Whether children have received training in painting at the rate corresponding to their preference for painting creation can also be judged (Questions 7 and 9 in the questionnaire). When children learn about different painting styles, they try to imitate the painting methods of these different painting styles; for instance, when children learn about abstract paintings, they try to change the shape of the objects in their paintings. From this aspect, Questions 4 and 8 can also assist in further judgments about whether children have received painting training or not. Therefore, the questionnaire can be used to screen children in terms of their eligibility to participate in the test. The questionnaire in this study was designed with relevant knowledge of the principles of painting creation. The questions in the questionnaire were all based on painting techniques and painting creation points. Another function of the questionnaire was to analyze children’s painting habits and to summarize the painting level of children of different ages. Furthermore, a part of the study comprised the digital image analysis of children’s paintings, in which MATLAB was used to convert all of the colored objects to black and to then calculate the ratio of the sum of all black pixels in the image to the total pixel value of the image. Lastly, children’s composition ability was judged based on the calculated results.

### 3.2. Participants

The participants involved in this study were children aged from 7 to 13 years old who had not received professional painting training. A total of 416 children participated in the test—182 girls and 234 boys. The children were divided into three age groups according to the characteristics of their painting learning (Table 1). All children were able to create paintings independently. Children who had not received professional painting training were involved in the test because some professionally trained children may have a fixed style in their paintings; as such, these children unconsciously use the composition method they have learnt when they creating paintings, and may create their own rules. Therefore, including children who have received professional training in the experiment would affect the results. In contrast, paintings by children who have not received professional painting training are influenced by various external factors; these type of paintings are based on their personal painting habits. Their paintings can help researchers identify the problems of painting techniques that children likely face in the creation process. Studying such children without professional painting training can help to determine painting characteristics and habits more effectively. All of the experiments in this study were conducted with the consent of the participant and the participant’s parents. None of the test results involve personal or private information. The study was conducted in accordance with the Declaration of Helsinki, and the protocol was approved by the Ethics Committee of Japan Advanced Institute of Science and Technology.

### 3.3. Data Preparation

The visual effect of a painting is best when the mathematical scale between the components in the painting is close or equal to 0.618. Some studies have shown that the golden ratio (Φ = 1.618033988749…) is a proportion that can evoke people’s sense of beauty; however, there is no related research on the value of the golden ratio [43]. Moreover, there are no research data showing that the golden ratio destroys the vision of beauty [44]. Therefore, the golden ratio can be considered the perfect proportion to create the best visual effects in painting. Furthermore, many of the artworks with good visual effects are in line with the golden ratio [45]. In this study, the value of the golden ratio was used to test the visual effects of children’s drawings. In research on the value of the golden ratio, it has been confirmed that artworks in the interval 0.606–0.63093 had a good visual effect [45,46]. When judging whether an object meets the golden ratio standard, the small difference between the actual calculation vale and the value of the golden ratio is acceptable [43,47]. Therefore, in this study, if the proportion of the main body of the paintings is in the range of 0.606–0.63093, it was judged that the proportion of the object of the painting work is appropriately arranged. If the ratio is less than 0.606, it was judged that the size of the object drawn by the child is small; however, if the ratio is over 0.63093, it was judged that the size of the object drawn by the child is large. The calculation of this ratio is based on Otsu algorithms in image binarization, which is the conversion of a color image into an image that is only black and white [14]. Converting objects drawn by children to black can be more effective or calculating the results. The Otsu algorithm automatically calculates the threshold based on the statistical properties of the entire image. Thus, this kind of algorithm is suitable for analyzing children’s paintings. 

**Figure 1:**
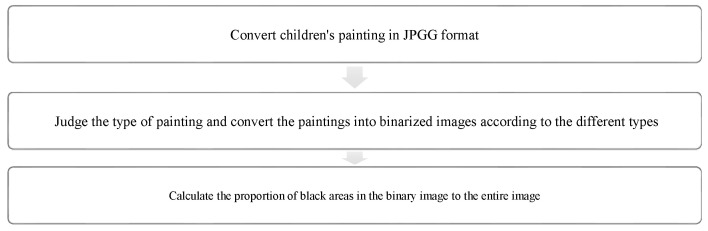
Flowchart of analysis of children’s paintings.

**Figure 2:**
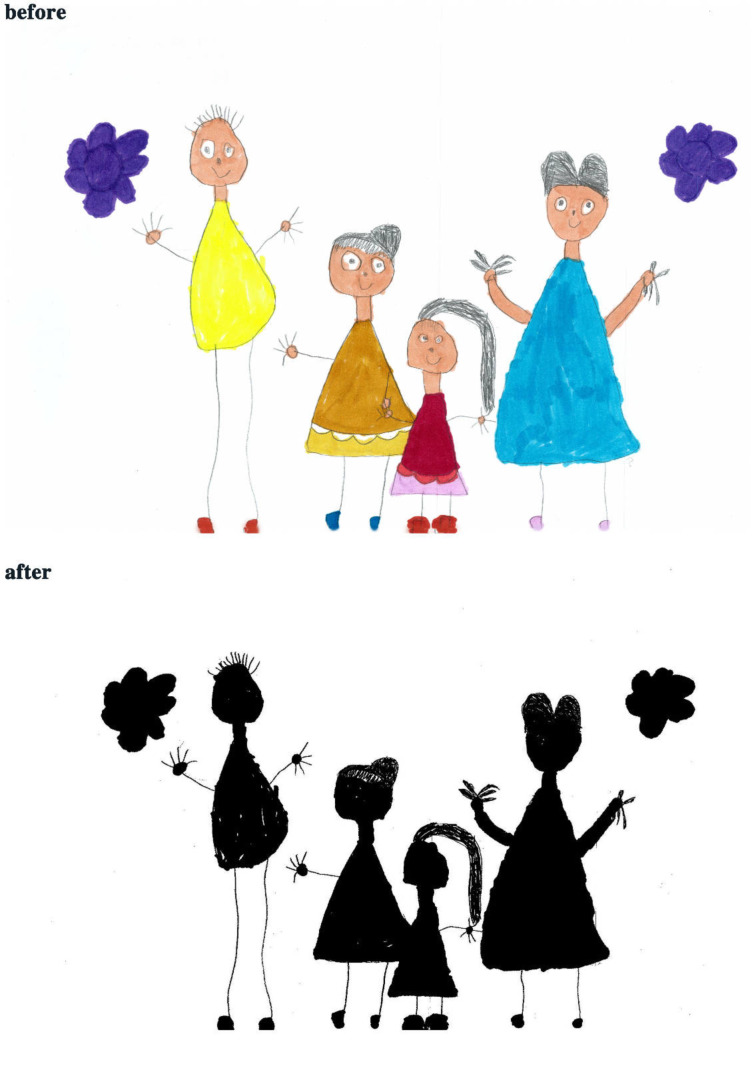
Example of a painting without a background color.

**Figure 3:**
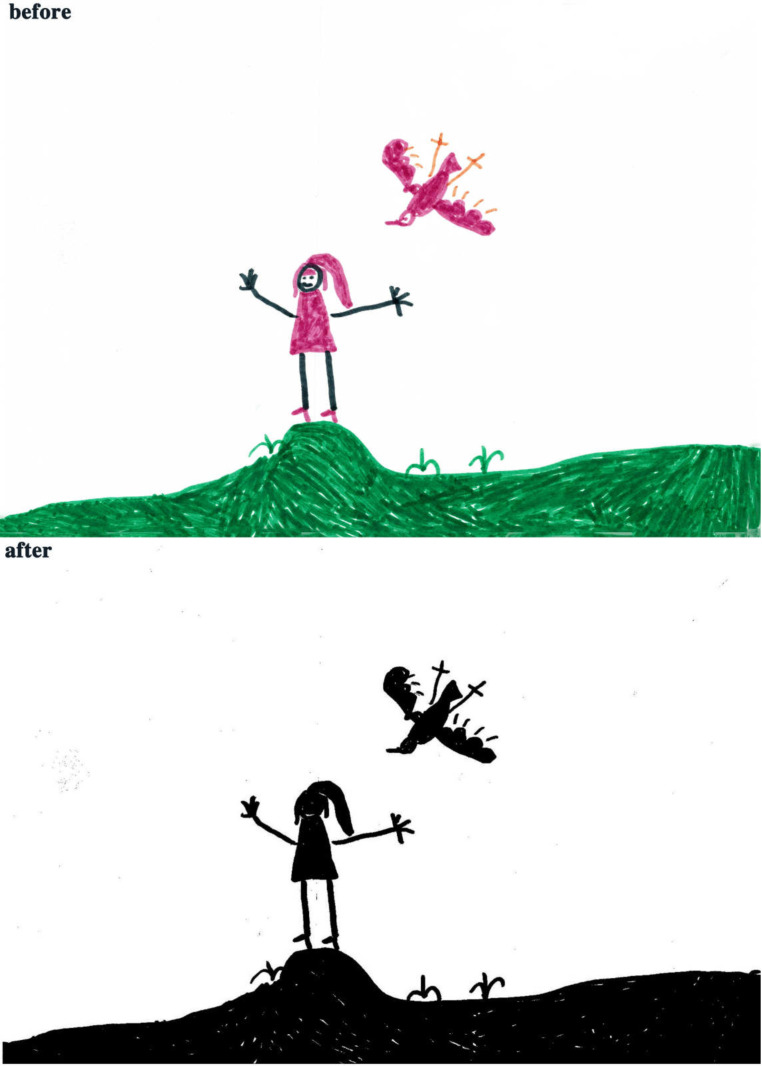
Example of a painting of a partial nature scene.

**Figure 4:**
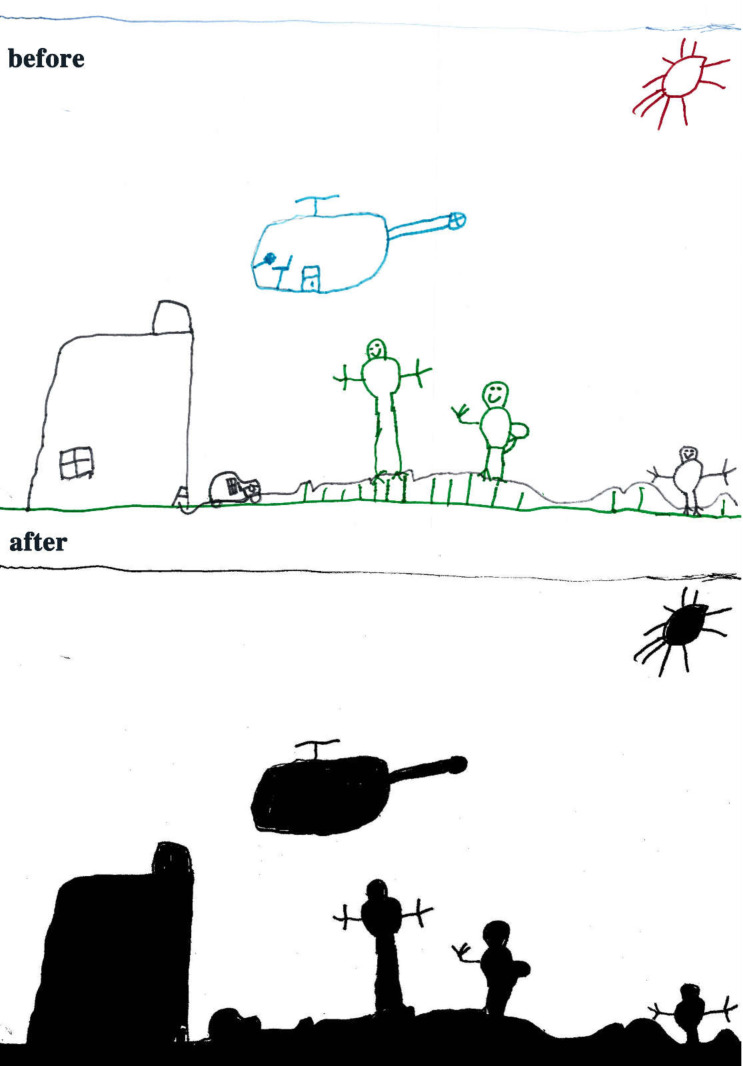
Example of a painting with lines only.

**Figure 5:**
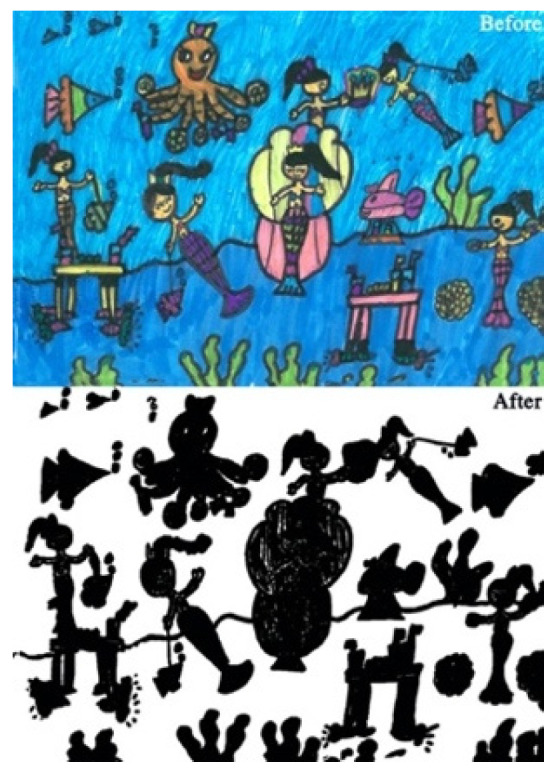
Example of a full composition painting.

**Figure 6:**
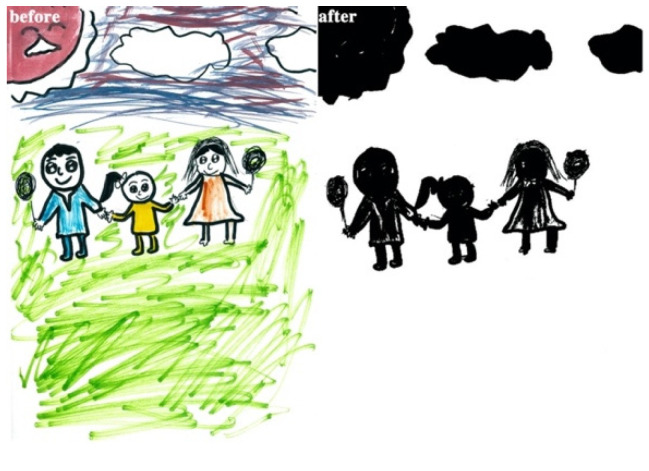
Example of a painting missing partial colors.

The study process of children’s paintings was divided into three steps (Figure 1). The first step was to convert children’s paintings in JPEG format. The resolution of all images used for analysis was 300 PPI. The second step was to judge the painting type and then to convert the paintings into a black and white image. The third step was to calculate the percentage of black pixels in the image as a percentage of the total number of pixels. Finally, based on the result of the calculation, it was judged whether the proportion of the subject in the child’s painting was appropriate. Before analyzing the proportion of subjects, the paintings needed to be pre-processed. Different children have different painting habits, and due to the differences in children’s composition methods and material usage habits, different composition forms will appear. Furthermore, different texture effects produced by different painting materials (such as colored pens, crayons, oil pastels, etc.) and painting papers would affect the study results; therefore, the painting materials used in children’s paintings were limited to colored pens and pencils (pencils were used for the contour lines of the objects in the paintings). The contour lines drawn with pencil were fine lines, and occupied only a small area of the entire painting. Therefore, paintings with pencils were also accepted. The papers that the children used were all white sketch paper. In order to improve the accuracy of the test, it was necessary to binarize the different types of paintings. According to the distribution of the objects in the paintings, the paintings of children who had not received painting training were divided into four types: Paintings without a background color, paintings with partial scene descriptions, paintings with lines only, and full compositions [2,48]. By sorting out children’s paintings, the following characteristics were observed: (1) Paintings without a background color (Figure 2 and Figure 3) lacked the depiction of an environment (such as the sky, clouds, sun, and background colors) and only simple characters or objects. In transforming these paintings, it was necessary to binarize the areas that represented the main content of the paintings; Figure 2 and Figure 3 show the features of this type of painting before and after image binarization. This test determined the proportion of the main content area of the painting to the overall painting work. (2) Some paintings were composed of lines. Therefore, when this type of painting was transformed, the area defined by the lines needed to be binarized. Figure 4 shows the effect of the binarization of images of this kind of painting. (3) Some paintings had a colored background (Figure 5). In this type of work, the paintings were full of color. If researchers convert an unprocessed painting to a binarized image, all of the colors in the painting will be binarized; thus, converting images in this way will affect the results of the test. Therefore, for this type of painting, the background color needed to be removed before image binarization. (4) Paintings that contained part of the background color (Figure 6) needed to have the background color removed before image binarization as it is not related to the main content of the painting.

### 3.4. Data Analysis

#### 3.4.1. Results of the Painting Analysis

**Figure 7:**
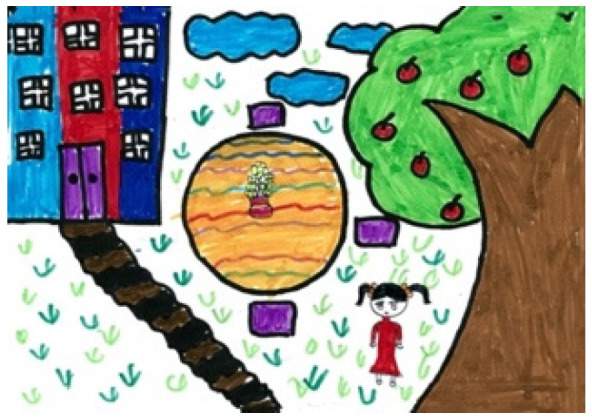
Example with an appropriate scale (0.606–0.63093) of objects.

**Figure 8:**
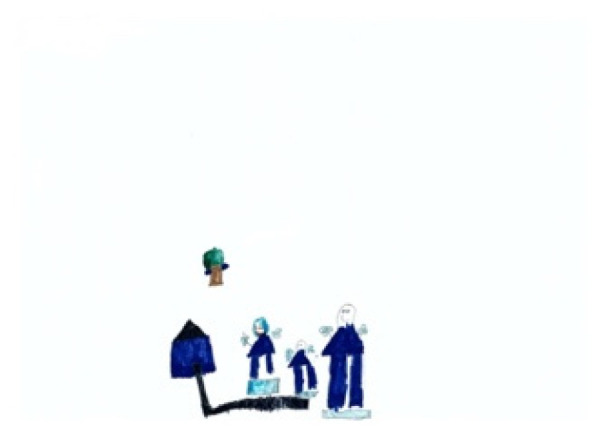
Example with a small scale of objects (less than 0.606).

**Figure 9:**
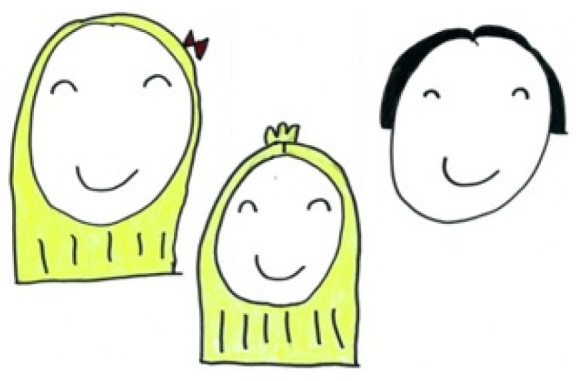
Example with a large proportion of objects (over 0.63093).

The painting data in this study were all analyzed by MATLAB. The statistical results of the scale of the objects in children’s paintings show that only 2.6% of them have an appropriate scale (Table 2). Figure 7 is a sample where the scale of the object in the painting is appropriate. Table 2 shows that 95.2% of children who had not received professional painting training used a small scale of objects in their paintings, and Figure 8 is a sample where the scale of the objects is in a small range. In addition, 2.2% of children who had not received professional painting training showed a large proportion of objects. Figure 9 is a sample where the proportion of the objects in the painting is large. Comparing the test results of boys and girls shows that the visual effects of girls’ paintings are stronger (the scale of the object) than those of boys’ paintings. The paintings of children aged 7–8 have the worst (arrange the objects in paintings with small scale) visual effects, whereas the visual effects of paintings for children aged 12–13 are the best (arrange objects in paintings with appropriate scale). Overall, most children aged 7–13 used a small scale of objects. The results show that girls draw more accurately than boys, and that children’s drawing ability develops with their growth.

#### 3.4.2. Results of the Questionnaire

The questionnaire data in this study were all analyzed using SPSS. From the questionnaire’s Cronbach’s alpha value (Table 3) of 0.913, it can be seen that the 416 children who participated in the questionnaire answered questions according to their situation. Therefore, the survey results of the questionnaire are reliable. In order to test whether the nine questions of the questionnaire truly express the characteristics of the variables studied, effectiveness analysis was performed using SPSS. From the results of Kaiser–Meyer–Olkin (KMO) tests (Table 3), it can be seen that the correlation between the nine questions in the questionnaire is strong.

The target testing group comprised the children who had not received professional painting training. In order to ensure that the participants met the requirement, the children answered the questionnaire before the painting test. The questionnaire determined the children’s knowledge of painting composition, technical terms of painting techniques, and the creation of paintings. If the child answered “no,” it indicated that they are not familiar with painting techniques and rules of painting creation, and have not received professional painting training. If they answered “yes,” they were assigned 0, and 1 otherwise. Table 4 shows the average score and median of each question in the questionnaire. Based on the average score of each question (which is higher than 0.7), the number of children answering “no” was higher than the number of children answering “yes.” The median value of each question in the questionnaire is 1; thus, more children answered “no” for each question. Therefore, the average score and median value of each question show that all children participating in the test met the test requirements.

Table 5 shows the impact test results of the position of the objects in the painting, characterization of the main part of the painting, the scene design, and the improvement in the shape of objects. Children’s attitude test result about the position of the objects is 0.357 (*t* = 9.104, *p* = 0.000 < 0.01), the characterization of the main part of the painting is 0.142 (*t* = 3.488, *p* = 0.001 < 0.01), the design is 0.268 (*t* = 6.573, *p* = 0.000 < 0.01), and the improvement of the shape of objects is 0.185 (*t* = 4.522, *p* = 0.000 < 0.01), which proves that these items have a positive impact on children’s attitudes toward painting creation. It also shows that the learning results of these four factors affect painting creation.

Regarding the test results of children’s ability to imitate paintings (Table 6), the *p*-values (two-tailed) of both test groups (boys and girls) were all higher than 0.05. Therefore, boys and girls had the same attitude toward the ability to imitate painting. The average result shows that the children who had not received professional painting training had a poor ability to imitate paintings. Additionally, in this direction, girls outperformed boys. Regarding the investigation of the layout of screen decoration (Table 6), the F statistic is 19.576, which corresponds to a probability *p*-value of less than 0.05. Thus, the population variances of the two measurements are significantly different. In addition, the *p*-values (two-tailed) of both test groups were less than 0.05. Therefore, there were differences between boys and girls in terms of the decorative layout of paintings. The average score shows that children who had not received painting training do not like to create paintings with fixed decorative styles. Boys do not prefer fixed decoration layout paintings more than girls do. The test results of children’s attitudes about painting creation show that the F statistic is 17.668 (Table 6), which corresponds to a probability *p*-value of less than 0.05. Therefore, the population variances of the two measurements are different. In addition, the *p*-values (two-tailed) values of both test groups were less than 0.05. The average scores for the tests of boys and girls were 0.80 and 0.71, respectively (Table 6). The average score shows that most children who had not received painting training think that creating paintings is difficult, and boys think that painting is more difficult than girls.

The correlation coefficient value of 0.575 (*p* = 0.00) shows that there is a significant positive correlation between the characterization of the main part of the painting and the size arrangement of the objects (Table 7). The characterization of the main part of the painting and the size arrangement of the objects are key in the effect of paintings. Both of these are in regard to the shape of the objects—there is a correlation between them. Since *t* = 3.29, and *p* = 0.00, there is a difference between two test items (the characterization of the main part of the painting and the size arrangement of the objects) in children’s cognition. In addition, the average value of Question 4 (0.78) is higher than the average value of Question 5 (0.71), which proves that children who have not received painting training pay more attention to the size arrangement of objects in painting creation.

We sought to determine the different attitudes toward the position of objects in the paintings; we explored the size arrangement of the objects and the attitude toward painting creation among children of different ages (Table 8). The test results are as follows. The *p*-value in Table 8 (*p-*values are all less than 0.05) shows that children of different ages have different perceptions of the location arrangement of objects and the size arrangement of objects and have different attitudes toward painting creation. The difference in the size arrangement of the objects in the paintings of children of different ages shows that the proportion of children aged 9–11who chose “yes” was 42.3%, which is significantly higher than the mean result of 28.85%. The difference in attitudes toward painting creation among children of different ages shows that 88.5% of children aged 9–11chose “no,” which is significantly higher than the average of 76.44%.

## 4. Discussion

### 4.1. Practical Importance

There are two main problems with children’s objects in paintings. First, most children who have not received professional painting training cannot control the scale of the objects; thus the proportion of objects in these children’s paintings is inappropriate. This problem also affects the visual effects of the children’s drawings. This problem arises because the children lack training in observation and composition. The training of children’s observation ability can be realized through analysis of fine arts. In the process of analyzing fine arts, children can understand the composition of a painting, the methods of painting creation, and the spatial relationship of objects in the painting. Another problem is the lack of decorative patterns in the paintings of children who have not received professional painting training. This problem results in a lack of ornamental value in children’s paintings; decorative patterns reduce the area of white space in paintings. These children’s observation abilities limit the creation of their paintings, and they do not know how to express the details of objects. Thus, a large area of white space appears in many children’s paintings. Therefore, teaching about imitation of decorative patterns in children’s drawing training can help children solve this problem. In training children to create decorative patterns in paintings, teachers can instruct them to add decorative patterns with simple geometric figures to their paintings. After a child has mastered a simple decorative pattern drawing method, teachers can guide them to change the shape of this decorative pattern as way to learn new patterns. This method can also diversify the decorative pattern styles in children’s paintings. As for proportion training, teachers can guide children to draw lines to divide their painting paper into different areas before they begin to paint. Additionally, teachers can add object observation courses to help children better understand the features of objects.

### 4.2. Theoretical Importance 

Imitation ability is the foundation for children to learn painting. However, in terms of creative behavior, children without painting training lack the ability to imitate paintings, and they cannot imitate painting work independently. Furthermore, the imitation ability of boys is lower than that of girls. The process of imitation training can help children to understand the process of painting creation, to improve their ability to use painting materials, and to learn about composition. Children can learn the creation process when imitating other people’s paintings, thus the process of learning to imitate is also the process of learning painting creation. Children can also improve their ability to use different painting materials by copying their works. Moreover, children who have not received painting training have a weak ability to create scenes of an environment in paintings. This problem is due to a lack of awareness of natural environments. To solve this problem, fine art teachers can lead children to sketch outdoors, which can help them to better observe and understand things in the natural environment.

## 5. Conclusions

The composition of a child’s painting determines its visual effects. In children’s painting practices, children can improve the visual effects of their paintings by considering the position and proportion of objects. Painting skills training can help children to improve their observation and esthetic abilities. According to digital image analysis, children who have not received painting training have a poor ability to control the scale of objects—the objects in these children’s paintings are small. Therefore, when guiding these children to create paintings, fine art teachers should pay attention to cultivating their ability to control the size of objects. The results of the questionnaire show that children who have not received professional painting training have a poor imitation ability. Children’s painting skills will be effectively improved after receiving imitation training, thus fine art teachers should deliver some imitation training. In addition, children who have not received professional training in painting think that the process of creating paintings is difficult, which is due to a lack of knowledge regarding painting composition and creative methods.

## Figures and Tables

**Table 1:** Gender and age distribution.

	Gender		Age			Total Number of Children
	Girls	Boys	7–8 Years Old	9–11 Years Old	12–13 Years Old	
***N***	182	234	191	129	96	416

**Table 2:** Results of the painting analysis.

	Less than 0.606	0.606–0.63093	Over 0.63093
Total (*N*) of children	396	11	9
Percentage	95.2	2.6	2.2
Boys	225	4	5
Girls	171	7	4
7–8 years old	189	1	1
9–11 years old	123	3	7
12–13 years old	84	7	5

Note: This is the proportion of black areas in the binary image to those of the entire image. Only 2.6% of children’s paintings have an appropriate scale; 95.2% of children who had not received professional painting training used a small scale of objects, and only 2.2% of children showed a large proportion of objects.

**Table 3:** Reliability and validity results. This study used the Cronbach’s alpha method to test reliability. The Kaiser–Meyer–Olkin (KMO) test result shows the validity of the questionnaire. The extraction method is principal component analysis. The results of the questionnaire are reliable.

Cronbach’s Alpha	0.913
Kaiser–Meyer–Olkin (KMO) measure of sampling Adequacy	0.907
*N* of items	9
Cases	416

**Table 4:** Average score and median results of the questionnaire. The median value of each question in the questionnaire is 1. The average score of each question is higher than 0.7.

Variables	Mean	Median
Characterization of the main part of the painting	0.71	1
Decoration layout	0.77	1
Imitation ability in painting	0.76	1
Characterization of the main part of the painting	0.78	1
Arrangement of the objects in painting creation	0.71	1
Scene design in the painting	0.77	1
Position of the objects	0.73	1
Improvement of the shape of the objects	0.80	1
Attitude about painting creation	0.76	1

**Table 5:** Impact test.

Model	*t*	Sig.
(Constant)	1.457	0.146
Position of the objects in the painting	9.104	0.000
Characterization of the main part of the painting	3.488	0.001
Scene design in the painting	6.573	0.000
Improvement of the shape of the objects	4.522	0.000

**Table 6:** Test of children’s imitation ability, decoration layout, and attitude toward painting creation. The *p-*values of these three items are all less than 0.05.

Variables	Mean		Std. Deviation		Std. Error Mean		Levene’s Test for Equality of Variance	
	Boys	Girls	Boys	Girls	Boys	Girls	F	Sig.
Imitation ability in painting	0.79	0.73	0.405	0.448	0.26	0.03	10.769	0.001
Decoration layout	0.81	0.71	0.395	0.453	0.26	0.34	19.576	0.000
Attitude about painting creation	0.80	0.71	0.398	0.453	0.26	0.34	17.668	0.000

**Table 7:** Paired samples test. The correlation is significant at the 0.01 level (2-tailed). The correlation coefficient value is 0.575 (*p* = 0.00). There is a significant positive correlation between the characterization of the main part of the painting and the size arrangement of objects.

Variables	Correlation	Mean	Sig. (2-Tailed)	*t*
Characterization of the main part of the painting	0.575	0.78	0.000	
Size arrangement of the objects in the painting	0.575	0.71	0.000	
Pair	0.575		0.001	3.289

**Table 8:** Attitude test among children of different ages.

Variables	Mean (%)	Age Group						Asymptotic Significance (2-Sided)		
		7–8 Years Old		9–11 Years Old		12–13 Years Old				
		No (%)	Yes (%)	No (%)	Yes (%)	No (%)	Yes (%)	Pearson Chi-Square	Likelihood Ratio	Linear-by-Linear Association
Position of the objects in the paintings	71.39	66.6	33.2	83.3	16.7	77.6	22.4	0.009	0.007	0.014
Size arrangement of the objects	76.44	75.7	24.3	57.7	42.3	67.2	32.8	0.006	0.008	0.025
Attitude toward painting creation	28.9	72.1	27.9	88.5	11.5	81.0	19.0	0.007	0.005	0.020

## Appendix A

Questionnaire

1. When you draw, do you like to arrange people or objects in a fixed position?
  Yes
  No
2. Do you like to decorate paintings with fixed patterns and colors?
  Yes
  No
3. Can you imitate other people’s paintings?
  Yes
  No
4. When you draw, do you like to emphasize an object or a person?
  Yes
  No
5. Do you think that the proportion, shape, distance, and direction of objects in paintings are important?
  Yes
  No
6. Do you feel it is difficult to create a picture of a scene (for example, an outing, a trip, a holiday, etc.)?
  Yes
  No
7. When you draw, do you care about the content of your work?
  Yes
  No
8. Can you change the shape of the objects in your paintings easily?
  Yes
  No
9. Do you like to create paintings?
  Yes
  No
